# Dual Graph Partitioning Highlights a Small Group of Pseudoknot-Containing RNA Submotifs

**DOI:** 10.3390/genes9080371

**Published:** 2018-07-25

**Authors:** Swati Jain, Cigdem S. Bayrak, Louis Petingi, Tamar Schlick

**Affiliations:** 1 Department of Chemistry, New York University, New York, NY 10003, USA; swati.jain@nyu.edu (S.J.); cigdem.sevim@gmail.com (C.S.B.); 2 Computer Science Department, College of Staten Island, City University of New York, Staten Island, New York, NY 10314, USA; Louis.Petingi@csi.cuny.edu; 3 Courant Institute of Mathematical Sciences, New York University, New York, NY 10012, USA; 4 NYU-East China Normal University Center for Computational Chemistry, New York University Shanghai, Shanghai 3663, China

**Keywords:** RNA graphs, dual graphs, graph partitioning, RNA substructures and submotifs, pseudoknots, ribosomal RNAs

## Abstract

RNA molecules are composed of modular architectural units that define their unique structural and functional properties. Characterization of these building blocks can help interpret RNA structure/function relationships. We present an RNA secondary structure motif and submotif library using dual graph representation and partitioning. Dual graphs represent RNA helices as vertices and loops as edges. Unlike tree graphs, dual graphs can represent RNA pseudoknots (intertwined base pairs). For a representative set of RNA structures, we construct dual graphs from their secondary structures, and apply our partitioning algorithm to identify non-separable subgraphs (or blocks) without breaking pseudoknots. We report 56 subgraph blocks up to nine vertices; among them, 22 are frequently occurring, 15 of which contain pseudoknots. We then catalog atomic fragments corresponding to the subgraph blocks to define a library of building blocks that can be used for RNA design, which we call *RAG-3Dual*, as we have done for tree graphs. As an application, we analyze the distribution of these subgraph blocks within ribosomal RNAs of various prokaryotic and eukaryotic species to identify common subgraphs and possible ancestry relationships. Other applications of dual graph partitioning and motif library can be envisioned for RNA structure analysis and design.

## 1. Introduction

The range of functions performed by ribonucleic acid (RNA) molecules in cellular processes—from protein synthesis [1] to gene regulation [2,3] and catalysis [4,5]—depends on their secondary (2D) and tertiary (3D) structures. The single stranded RNA chain folds upon itself to form double-stranded helical and single-stranded loop regions. These 2D structural elements interact with one another to create functional 3D structures. RNA molecules tend to fold in a hierarchical manner [6,7], with complex 3D structures consisting of substructures or submotifs. Such submotifs often correlate with specific function. Common submotifs between two RNAs can thus suggest functional or evolutionary relationships. Understanding the structure/function relationships of RNA molecules is crucial for manipulating their functions and for designing novel RNA molecules for various industrial and therapeutic applications [8,9].

With the growing number of RNA structures solved experimentally, computational modeling of RNA structures for analysis, prediction, and design becomes increasingly important [10]. A common approach to simplify RNA models is to represent them using coarse graining (see review [11]) that reduces the relevant number of degrees of freedom. Such approaches can offer systematic structure analysis that can help connect structure to function and pursue design applications. Coarse-grained models often represent RNA nucleotides as single or multiple beads [12,13,14,15,16,17,18,19], which are then sampled by various techniques to generate RNA conformations or ensembles. Another type of coarse-grained representation involves graph theoretical approaches for the study and analysis of RNA 2D structure [20,21,22].

**Figure 1:**
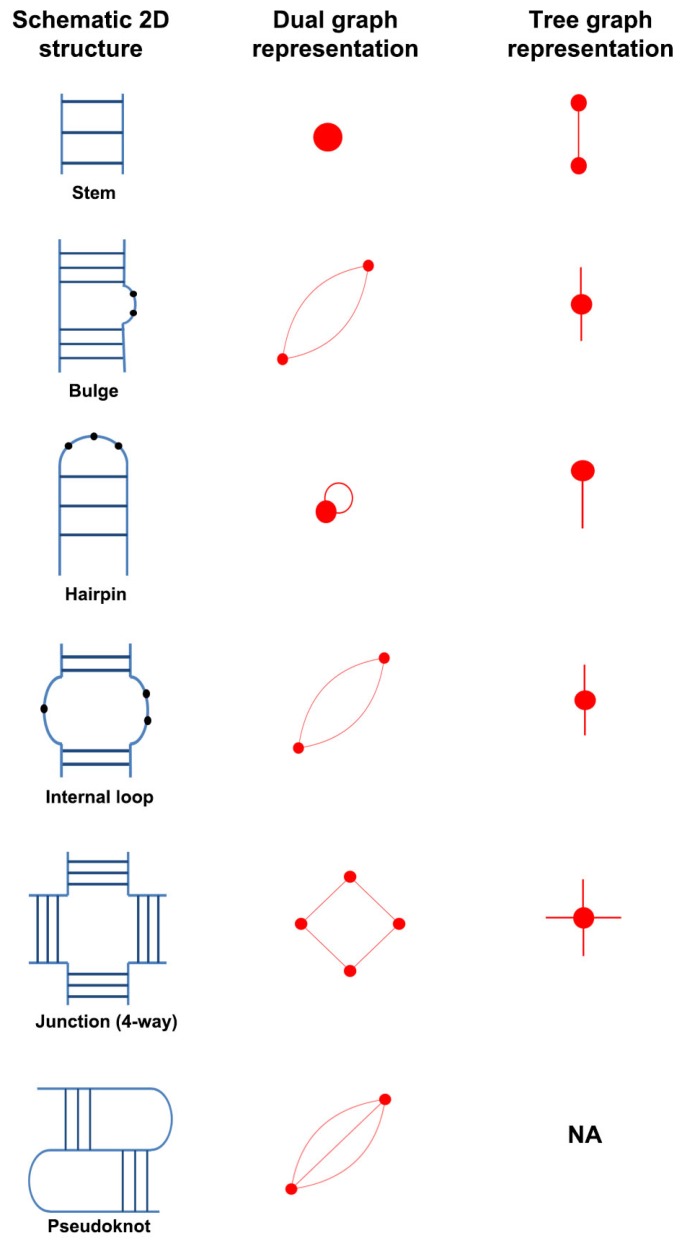
Dual graph representations of common RNA 2D structure building blocks. In dual graphs, stems with at least two base pairs are denoted by vertices. All loop strands (with or without residues) are represented as edges, except for single-residue bulges and internal loops with only one residue in each strand, which are ignored. The tree graph representations are also shown for comparison. Note that pseudoknots cannot be represented by tree graphs (indicated by Not Applicable (NA)) because they contain intertwined and non-nested base pairs.

RNA 2D structures were first represented as graphs by Waterman [23], followed by Nussinov [24,25] and Shapiro [26]. Recently, graphs were used to represent individual residues along with their local and long-range RNA interactions and search for common interaction patterns in RNA structures [27]. Our RNA-As-Graph (RAG) framework represents RNA 2D structures as tree and dual graphs by translating RNA stems (or helices) and loops into edges and vertices based on simple rules [28,29]. Tree graphs represent the single stranded 2D elements (e.g., hairpins, internal loops, bulges, and junctions shown in Figure 1) as vertices, and double stranded stems as edges; dual graphs represent the single stranded 2D elements as edges and stems as vertices. Apart from a lower number of degrees of freedom, graph representation can also make possible the use of graph theory methods to enumerate RNA 2D structures, as well as design and predict 3D structures (see reviews [8,9,22]). Graph representation of RNA structures also lends itself to the modularity and hierarchical architecture of RNA structures through graph partitioning into subgraph building blocks [30,31,32,33]. Those subgraphs can be utilized to design sequences and build atomic models that fold onto novel RNA motifs (identified by clustering techniques) [34,35,36]. To date, RAG has been used to analyze, classify, predict, and design RNAs [34,35,36,37,38,39,40,41], and catalog tree graph topologies up to 13 vertices (where vertices denote loops) [36] and dual graph topologies up to nine vertices (where vertices represent stems) [42].

The tree graph representation is intuitive and convenient in many applications, most recently in structure prediction [41] and design [35]; however, tree graphs cannot represent more complex RNA features like pseudoknots, namely regions of intertwined and non-nested base pairing (see Figure 1) as present in many RNAs. RNA pseudoknots are biologically important and common in nature but often excluded in computational studies due to their complexity. Because pseudoknots are essential for the function of many RNAs (e.g., riboswitches and viruses) [43,44,45], handling them is not only important for analyzing RNA structure and function, but for RNA design as well. The more general dual graph objects contain self loops and multiple edges between two vertices and have an advantage of representing pseudoknot topologies [28].

Graph theory offers natural ways to divide RNA 2D structure modeled as graphs. Previously, we have applied a mathematical graph partitioning approach to segment tree graphs [31]. We also developed a more biological partitioning approach [46] for motif search, structure prediction, and design [35,41]. Recently, we reported a graph-partitioning algorithm for dual graphs [47,48] that divides a graph into non-separable subgraphs, termed *blocks*. A block is a subgraph whose vertices cannot be removed to produce disconnected graphs or a graph composed of a single vertex. The dual graph partitioning algorithm helps identify all blocks while keeping all RNA junctions as well as pseudoknots intact.

Here, we report an RNA submotif library of RNA substructures represented as dual graphs by partitioning the 2D structures of a representative set of 2280 RNA structures (obtained from the Bowling Green State University (BGSU, Bowling Green, OH, USA) RNA website, see Section 2.4) using our dual graph partitioning algorithm [47,48]. We identify 94 different dual graph topologies (between 2 and 9 vertices) that represent experimentally solved RNA structures. These topologies correspond to our list of dual graph motifs for existing RNA structures (available online [49]). By applying our dual graph partitioning algorithm to this representative set of RNA structures, we identify 56 dual graph topologies (between two and nine vertices) as non-separable subgraph blocks. Of the 56 dual graph topologies, only 22 have five or more occurrences in our representative dataset. Among these 22 recurring motifs, 15 contain pseudoknots. Internal loops, junctions, and small motifs with pseudoknots emerge as the most common submotifs.

We further extract 3D atomic fragments for all examples corresponding to different subgraph blocks in our representative RNA dataset, and catalog them based on their dual graph ID numbers to create a dataset of RNA 3D substructures, which we call *RAG-3Dual*. The dataset contains a total of 5332 atomic fragments corresponding to the 56 subgraph blocks (between 2–9 vertices), and 48 atomic fragments for dual graph blocks with ≥10 vertices. RAG-3Dual serves as a first step in using dual graphs and associated atomic components to analyze and search substructures among RNAs, and for 3D structure prediction and design of RNAs by fragment assembly (similar to the tools we developed for tree graphs [35,41,46]).

As an application of graph partitioning, we analyze the distribution of submotifs within ribosomal RNAs (rRNAs) of various prokaryotic and eukaryotic species in our representative dataset. Our analysis reveals subgraphs that are present in all species we analyzed and subgraphs that are common to a subset of species (and may relate to evolution), as well as subgraph blocks that are exclusively present in rRNA structures. These findings suggest that graph partitioning may be useful for evolutionary analysis and that some submotifs may be unique to rRNAs. Further applications of our dual graph work for motif search and design can be envisioned.

## 2. Materials and Methods

In this section, we describe the methods and data we use for the extraction, partitioning, and analysis of RNA submotifs using dual graphs.

### 2.1. RNA Dual Graph Representation

RNA molecules are chains made up of four nucleotides: adenine (A), guanine (G), cytosine (C), and uracil (U). RNA chains can fold onto themselves to form canonical base pairs (GC, AU, and GU wobble). These double-stranded regions are called *stems or helices*, and the single-stranded regions connecting the helices are called *loops* (e.g., hairpins, bulges, internal loops, and junctions). The secondary (2D) structure describes the connectivity between various stems and loops in an RNA chain. A common 2D structural motif found in RNA chains is a *pseudoknot*, which is composed of intertwined base-pairing regions that are not well nested (Figure 1).

Our RAG resource represents the 2D structure of RNAs as dual graphs using the following rules [28,29]:

1. An RNA stem (or helix) with at least two canonical base pairs is considered as a vertex.
2. Each loop strand between two helices is denoted as an edge. Single-residue bulges and internal loops with only one nucleotide in each strand are ignored.
3. Uninterrupted hairpin loops (including helical ends) are represented as self loops.
4. Unpaired bases or helical ends at the 5${}^{\prime }$ and 3${}^{\prime }$ ends of RNA molecules are not represented.

Figure 1 shows dual graph representations of common RNA 2D structural elements. Tree graph representation for these 2D structural elements are also shown. Apart from the definition of vertices and edges being reversed, the main difference between tree and dual graphs is that the latter can contain cyclic paths. That is, a path can have the same start and end vertex without repeated edges. This feature allows dual graphs to represent more complex structures like pseudoknots.

The usage of graphs to represent RNA 2D structures reduces the degrees of freedom and provides a quantitive way to study RNA topology. For a given graph G=(V,E), where *V* is the set of vertices and *E* is the set of edges, the Laplacian matrix, a mathematical representation of graph/network connectivity, is defined as L=D−A. Here, *D* is the degree matrix and *A* is the adjacency matrix. *D* specifies the number of edges at each vertex, i.e., degree of a vertex as diagonal entries $d_{i}$, i=1,…,n. The matrix *A* indicates the number of edges between each pair of vertices, i.e., $a_{ij}=$ number of edges between *i* and *j*, 0 if *i* and *j* are not connected; $a_{ii}=2$ if there is a self loop at the vertex *i*, 0 otherwise. By construction, the diagonal element $d_{i}$ of matrix *D* is equal to the sum of all elements in row *i* of matrix *A*. Self loops are ignored when calculating the Laplacian.

The eigenvalue spectrum of *L* is a measure of graph connectivity, and can help us to classify RNAs based on our graph representations. For a dual or tree graph of *n* nodes, the *n* eigenvalues of *L* are non-negative real numbers, as *L* is symmetric. The first eigenvalue, $\lambda_{1}$, is always zero. The second smallest eigenvalue, $\lambda_{2}$ (also known as the Fiedler eigenvalue), measures the algebraic-connectivity or the compactness of a graph topology [50]. Graphs with similar $\lambda_{2}$ values tend to have similar topologies [28,29]. In addition to eigenvalues, eigenvectors also contain useful information about the graph; for example, the second eigenvector, $\mu_{2}$ (corresponding to $\lambda_{2}$) provides information about the connectivity of a graph, and can be used to partition a tree graph (and associated RNA 2D structure) in various ways into topologically distinct components, as we have done in [31].

### 2.2. Dual Graph Enumeration

Our dual graph definitions as described in Section 2.1 carry the following implications. Any RNA helix is connected to other helices by four strands (two incoming and two outgoing, with self loops counted twice), with the exception of the helix (or helices) at the 5${}^{\prime }$ and 3${}^{\prime }$ ends. If the 5${}^{\prime }$ and 3${}^{\prime }$ ends are on the same helix, then that helix only has two outgoing strands; if the 5${}^{\prime }$ and the 3${}^{\prime }$ ends are on different helices, then both have three incoming and/or outgoing strands. Therefore, all dual graphs representing RNA structures have one of the following degree sequences: (4, …, 4, 2) or (4, …, 4, 3, 3). Using the above rules and a probabilistic graph growing method, we have previously generated dual graphs up to nine vertices in our graph library [29,34]. In each graph growing cycle, two vertices are selected from the set of *V* vertices, and are connected by randomly selected number of edges (one, two, or three). Because the previous vertex is always selected from the connected graph (except for the first step), all enumerated graphs are inherently connected. Dual graphs with the same Laplacian spectrum are removed. The complexity and number of dual graphs increase rapidly with vertex number, and hence it may not be possible to exhaustively enumerate all possibilities for larger values of *V* [29].

The dual graphs in our library are cataloged based on the number of vertices and the eigenvalue spectrum of the corresponding Laplacian. Specifically, each dual graph ID has the form V_n, where *V* is the number of vertices and *n* is a unique integer (based on the Laplacian spectrum) which distinguishes among the dual graphs with the same number of vertices. For vertex numbers *V* = 2, 3, 4, 5, 6, 7, 8, and 9, our enumerated dual graph library contains 3, 8, 30, 108, 494, 2388, 12,184, and 38,595 graphs, respectively [34]. The annotation for these dual graph topologies (as either existing, RNA-like, or non RNA-like) was last performed in 2011 [42]; dual graphs were not included in our more recent update for tree graph annotations [36]. Although we have not annotated existing graphs for our entire dual graph library, we have annotated dual graphs corresponding to the representative set of RNA structures used in this work; see Section 2.4 and Section 2.5.

To assign a dual graph ID to a given RNA structure, we convert the 2D structure to a dual graph using the above rules (Section 2.1), calculate its Laplacian and the corresponding eigenvalue spectrum, and compare that to those that exist in our library. If the eigenvalue spectrum of the query RNA is a positive match to any of the dual graphs in our library, its graph ID is assigned to the RNA [29]. Only the vertex number is reported for dual graphs with < 2 or > 9 vertices. Because the dual graph representation is coarse-grained and does not take into account the number of residues in stems and loops, the same graph topology can correspond to multiple RNAs (i.e., with two or five base pairs in a stem or different number of bases in a loop).

### 2.3. Dual Graph Partitioning Algorithm

Simplified graph representations of RNAs provide a systematic and efficient approach for partitioning them into topologically and biologically meaningful submotifs. Our recent partitioning method for dual graphs maintains pseudoknots and junctions intact [47,48]. The algorithm applies the John Hopcroft and Robert Tarjan algorithm [51] for identifying non-separable graph components in a connected graph. It takes the adjacency matrix of the graph representation as input and determines articulation points. A vertex *v* is said to be an articulation point if G−v results in a disconnected graph, or if a single vertex remains. Furthermore, if a graph (or subgraph) does not have an articulation point and cannot be divided further, it is called non-separable i.e., a *block*. A block corresponds to a pseudoknot if it contains a vertex *v* of degree at least three i.e., three or more edges are incident at *v*, not counting self loops [47,48]. The dual graph partitioning code is available online [52].

**Figure 2:**
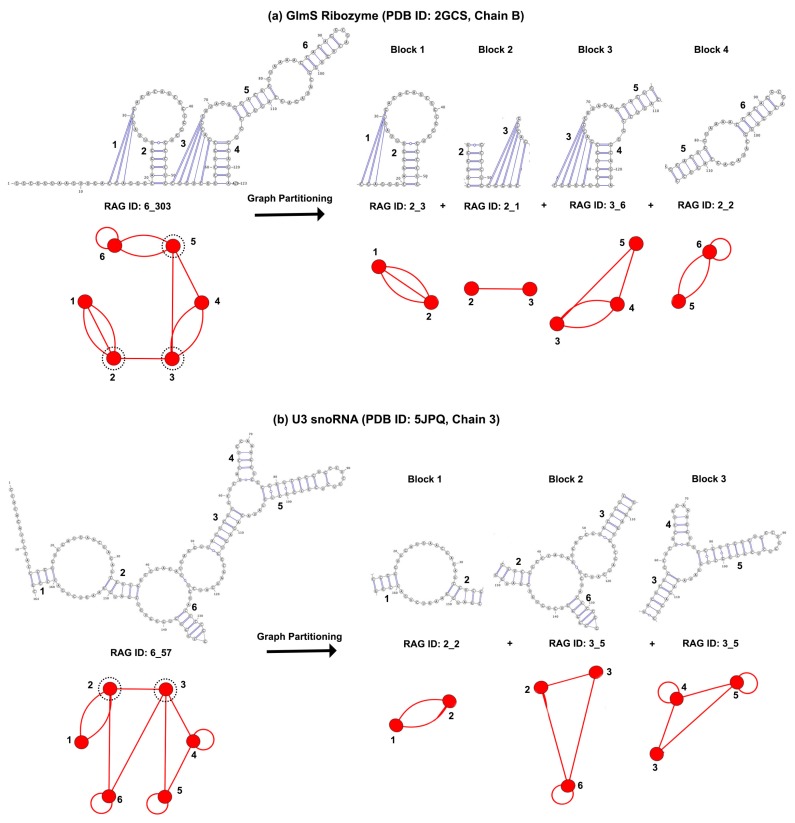
Dual graph partitioning. Illustration for (**a**) structure with pseudoknots; and (**b**) structure with junctions. Dashed black lines show the articulation points, where the graph will be cut.

In Figure 2, we illustrate the graph partitioning on two sample structures, one containing pseudoknots and one containing junctions. The articulation points that partition the graph are marked with dashed lines. As designed, our partitioning does not separate the two strands of the pseudoknots, and maintains all helices connected to a junction in the same subgraph block. Following partitioning, the vertices corresponding to each subgraph block are extracted, and new Laplacian matrices and corresponding eigenvalue spectra are calculated to assign them dual graph IDs (as described in Section 2.2). Note that, according to our definitions (Section 2.1), the presence of self loops in the dual graph or subgraph does not affect the graph ID assignment (as self loops are ignored when calculating the Laplacian); RNA structures and substructures of the same topology but different number of hairpin loops have the same graph ID (e.g., in Figure 2, block 4 of the GlmS ribozyme and block 1 of the U3 small nucleolar RNA (snoRNA) are both graph topologies 2_2, and blocks 2 and 3 of U3 snoRNA are both graph topologies 3_5).

### 2.4. Representative Set of RNA Structures

The representative set of RNA structures obtained from the BGSU RNA site available online in [53] is used in this study. This website lists representative RNA 3D structures from all RNA structures deposited in the Protein Data Bank (PDB) according to the methodology described in [54]. In this list, RNA 3D structures are grouped into equivalence classes based on sequence, structure, and species, and one structure from each equivalence class is selected as a representative. The list is updated automatically each week. We used the representative set with ID: 2.151, released on 21 October 2017, containing 2280 equivalence classes. (For reference, a recent update with ID: 3.11, released on 1 March 2018, contains 2354 equivalence classes.)

RNA 3D structures listed in the representative set were downloaded from the PDB (including multiple PDB format files for large structures in mmCIF format), and RNA chains listed in the set were extracted. Duplicate entries within a single equivalence class (duplicate chains and multiple NMR models) were removed, along with water and ligand molecules. Modified bases were retained, and the residues were renumbered if the PDB file contained insertion codes (residue numbers containing letters). Chains from a single PDB file separated into different equivalence classes were not combined. The RNAView program [55] was used to determine corresponding 2D structures. Only canonical base pairs were retained, with preference given to AU and GC Watson–Crick base pairs over the GU wobble base pair. Files with no base pairs, only isolated single base pairs, or single stems were removed. Note that such cases lead to at most one-vertex dual graphs, which have no corresponding topology nor adjacency matrix. The remaining 863 of the 2280 2D structure files were used as input for dual graph representation and partitioning.

### 2.5. Defining Existing Dual Graph Topologies

**Figure 3:**
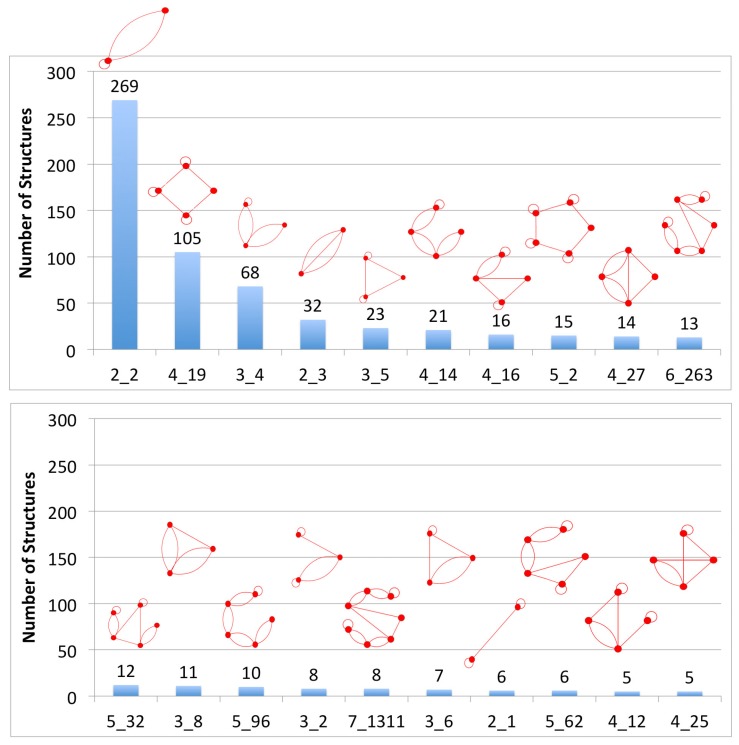
Common existing dual graph topologies. Existing dual graph IDs with five or more structures in the representative dataset of RNA structures are shown. See Table S1 in the Supplementary data for complete details of all 94 existing dual graph topologies.

We determined dual graphs for 863 2D structures in the representative list of RNA 3D structures. Of these, the dual graphs for 108 structures contained ≥ 10 vertices. For these 108 structures, there are no dual graph IDs in our library since we have enumerated only dual graphs between two to nine vertices. For the remaining 755 structures, we identified 94 unique dual graph topologies. These include four new topologies with nine vertices that correspond to RNA structures but did not exist in our enumerated dual graph library (Section 2.2). They are: 9_38596—viral IRES RNA, 9_38597—FMN riboswitch, 9_38598—18S rRNA fragment, and 9_38599—U2 and U6 human snRNA. We have added these new topologies to our dual graph library. Of the 94 topologies, only 20 topologies correspond to five or more structures, and are shown in Figure 3. The most common dual graph topologies are 2_2 that corresponds to two stems with an internal loop, and 4_19 that corresponds to a 4-way junction. See Table S1 in Supplementary data for details and images of all 94 topologies and Table S2 for number of structures with dual graphs ≥ 10 vertices.

Next, we updated dual graph topologies classified as existing topologies (i.e., dual graph topologies corresponding to RNAs found in nature) in the 2011 update to our RAG resource [42]. Previously, we had used information from three main sources to construct RNA dual graphs for 2D structures: the Rfam database (contains consensus 2D structures of RNA families), Pseudobase++ database (catalog of RNA pseudoknots), and the RNA Strand database (contains 2D structures from the PDB, Signal Recognition particle (SRP) database, and others); any RNAs with > 200 nucleotides or any synthetic RNAs were not considered. This had led to the classification of 71 dual graph topologies as existing. Since then, we have redefined our definition of existing graph topologies to include only those RNA structures that have been fully experimentally validated (i.e., RNAs with available 3D structures) for classifying tree graph topologies [36]. Therefore, we now classify the 94 dual graph topologies (with 2–9 vertices, listed in Table S1 in the Supplementary data) in the representative RNA dataset as existing dual graph motifs; note that this representative dataset of RNA structures (see Section 2.4) may still miss some existing topologies. These classified topologies, along with associated RNA structures (PDB IDs and chains from the dataset used here), are available online [49] (see Supplementary material S2 for a description of the dual graph resource).

Comparing the new set of 94 dual graph topologies to the 71 we classified as existing in 2011, we find that 34 are common. Among the 37 of 71 that are missing, 24 topologies corresponded to RNA 2D structures from the Rfam, Pseudobase, or the SRP database; and 13 were from the PDB but with duplicate chains, chains from different RNA molecules separated in the representative dataset, or segments of RNA molecules part of larger RNA structures in the representative dataset. Therefore, these 37 dual graph topologies (listed in Table S3 in Supplementary data) were not considered in the set of existing topologies.

## 3. Results

### 3.1. Partitioning Dual Graphs into Subgraphs

**Figure 4:**
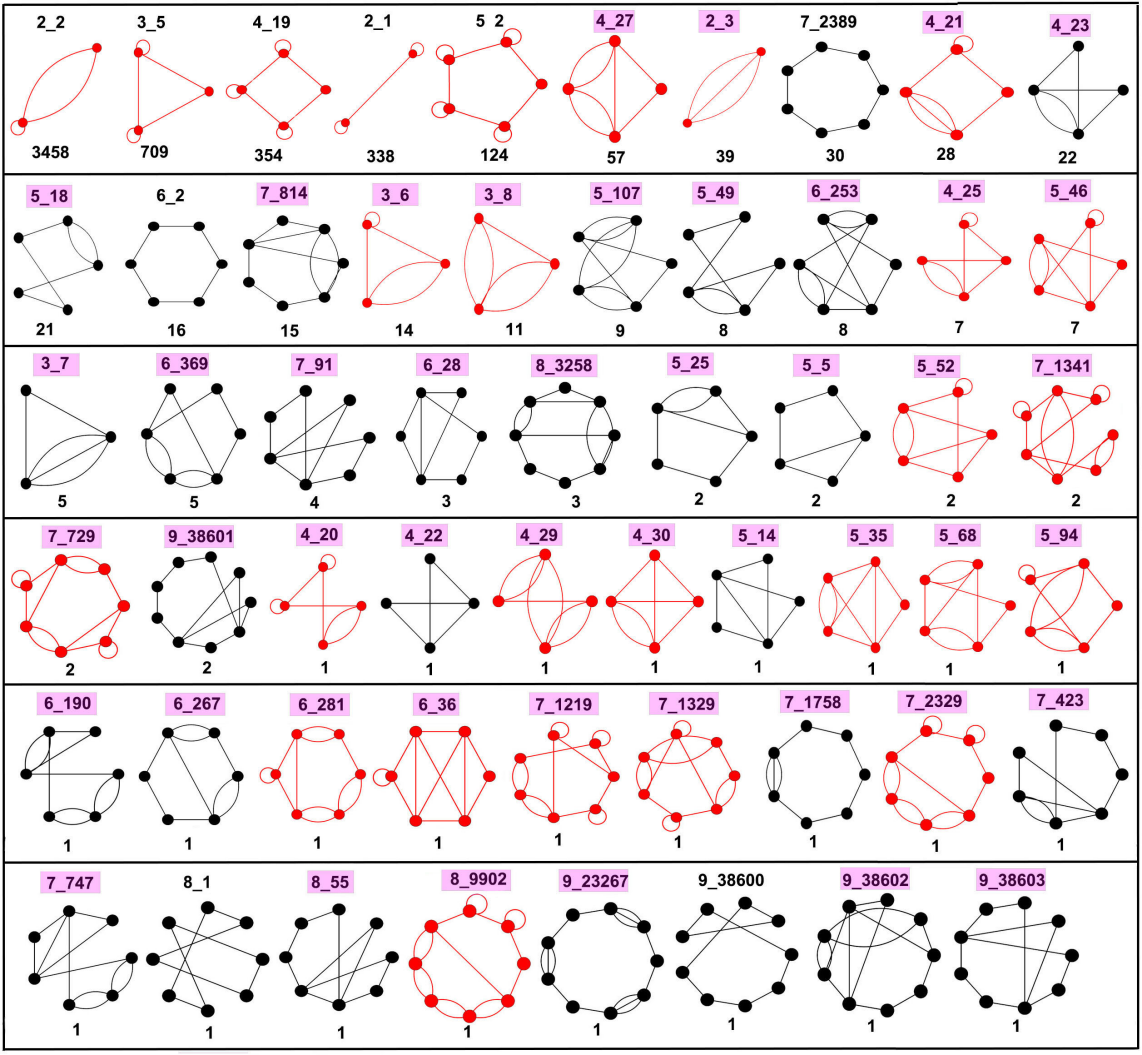
Subgraph block topologies with 2–9 vertices in decreasing order of occurrence frequency. The 56 subgraph block topologies with 2–9 vertices in the representative dataset of RNA structures are shown, along with graph IDs and number of occurrences. Red graphs are part of existing dual graph topologies corresponding to the representative RNA 3D structure dataset (Figure 3 and Supplementary Table S1). Black graphs are those that only emerge as subgraphs. The IDs of subgraphs with pseudoknots are highlighted in magenta. Subgraphs with 10 or more occurrences are shown in Figure 5 (pseudoknot-free) and Figure 6 (with pseudoknots). Atomic fragments corresponding to these 56 subgraph blocks are cataloged in the RAG-3Dual dataset of RNA 3D substructures.

Now that we have our dual graph library and representative dual graphs, we analyzed the RNA submotifs (in the form of non-separable dual graph blocks) by applying dual graph partitioning algorithm (as described in Section 2.3) to the 863 structures with more than one vertex in their dual graph representation. This produced 56 different dual graph topologies of 2–9 vertices that emerge as subgraph blocks, as shown in Figure 4. Of the 56 graph topologies, 27 (colored red) are also part of the 94 existing dual graph topologies (Section 2.5), and the remaining 29 (colored black) emerge only as subgraph blocks of larger graphs after partitioning. The 56 dual graph block topologies also include five new topologies that emerged as subgraphs but did not exist in our enumerated dual graph library (Section 2.2). They are: one topology with seven vertices (7_2389) corresponding to a 7-way junction present in ribosome structures, and four topologies with nine vertices: 9_38600 present in *Trypanosoma cruzi* 60S ribosomal subunit, 9_38601 present in RNAse P RNA, 9_38602 present in B12 binding riboswitch, and 9_38603 present in group II intron. We have added these new topologies to our dual graph library.

Of the 56 subgraph topologies, only 15 have 10 or more occurrences in the representative dataset, and only 22 have five or more occurrences (first three rows in Figure 4). Of these 22 subgraph topologies, 15 contain pseudoknots. Furthermore, of the total of 5380 subgraph blocks found in 863 structures, 97.7% of the subgraph blocks contain between 2–6 vertices, 1.3% contain between 7–9 vertices, and about 1% are larger subgraphs with more than nine vertices. These results suggest that most RNAs are composed of a small number of subgraph topologies. Furthermore, complex RNAs could be assembled from relatively simple components.

**Figure 5:**
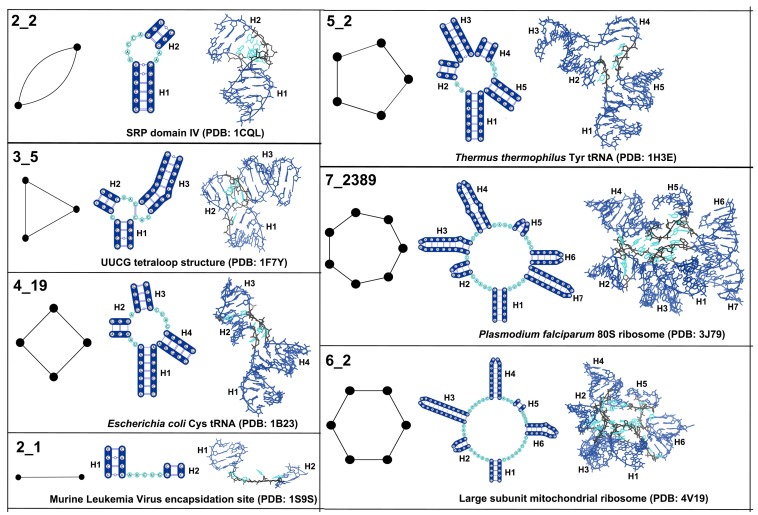
Common subgraph blocks without pseudoknots. Shown for each dual graph block topology with 10 or more occurrences (found by partitioning dual graphs in the representative RNA dataset) are the 2D and 3D structure fragments of one representative example. The same colors in the 2D and 3D structures correspond to similar regions. Helices are marked as *Hi*, where *i* indicates the helix number.

Figure 5 shows the 2D and 3D structures of one example for top dual graph blocks (with 10 or more occurrences) without pseudoknots. These topologies correspond to internal loops (2_2), 3-way junctions (3_5), 4-way junctions (4_19), two connected stems (2_1), 5-way junctions (5_2), 7-way junctions (7_2389), and 6-way junctions (6_2), respectively. These top topologies correspond to some of the most common motifs in RNA structures.

**Figure 6:**
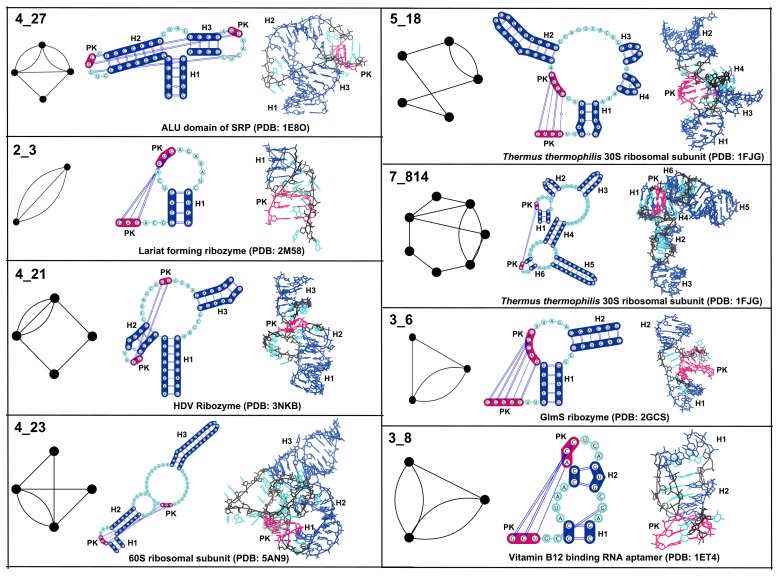
Common subgraph blocks with pseudoknots. Shown for each dual graph block topology with 10 or more occurrences (found by partitioning dual graphs in the representative RNA dataset) are the 2D and 3D structure fragments of one representative example. The same colors in the 2D and 3D structures correspond to similar regions. Pseudoknots are marked as *PK* and are shown in magenta. Standard helices are marked as *Hi*, where *i* indicates the helix number.

Figure 6 shows examples corresponding to the top graph blocks (with 10 or more occurrences) that contain pseudoknots. These topologies correspond to 4_27 (e.g., 3-way junctions with a pseudoknot between two hairpin loops), 2_3 (two helix pseudoknot), 4_21 (e.g., 3-way junction with pseudoknot between hairpin and junction loop residues), 4_23 (e.g., pseudoknot between two internal loop residues), 5_18 (e.g., 4-way junction with a pseudoknot), 7_814 (e.g., pseudoknot between 3-way and 4-way junction), 3_6 (three helices with pseudoknot involving internal loop residues), and 3_8 (three helices with pseudoknot involving hairpin loop residues). Most of the commonly known RNAs with pseudoknots contain one of the above mentioned pseudoknot motifs: A and G riboswitches contain the submotif 4_27; the twister ribozyme contains the simplest pseudoknot submotif 2_3; HDV ribozymes contain the submotif 4_21 (some structures also contain a less frequent 5_52 submotif); 3_6 submotif is present in GlmS ribozyme, SAM riboswitch, fluoride riboswitch, and preQ1 riboswitch; and 3_8 motif is common in HIV RNA structures. Subgraphs 5_18 and 7_814 are present exclusively in ribosome structures (see Section 3.2). The tetrahymena ribozyme and Hepatitis C virus internal ribosome entry site (HCV IRES) domain contain the less frequently observed pseudoknot motifs of 5_5 and 5_25, respectively.

The same dual graph motifs are also found in unrelated RNA structures. For example, the 3_6 graph motif is found in inhibitor bound GlmS ribozyme (example shown in Figure 6) as well as in box H/ACA RNA and viral IRES RNA. Similarly, the 3_8 graph motif is found in HIV dimerization initiation sites, where it represents kissing hairpin loop motifs; we also find it in vitamin B12 binding RNA aptamer (example shown in Figure 6) and SAM-II riboswitch, and it represents pseudoknots formed between dangling end and hairpin residues in both structures.

Based on the dual graph partitioning results, we have extracted the atomic fragments of different subgraph blocks from the corresponding PDB files, and assembled a dataset of RNA 3D substructures, which we call *RAG-3Dual* (available online [49]). The atomic fragments (along with their PDB IDs, chain, and residue numbers) are cataloged based on their subgraph IDs. RAG-3Dual contains a total of 5332 atomic fragments corresponding to the 56 subgraph blocks shown in Figure 4, and 48 atomic fragments for dual graph blocks with ≥ 10 vertices (these are not assigned graph IDs, see Table S4 in Supplementary data). This serves as a first step in constructing a library of atomic substructures that can be used for searching similar substructures, 3D structure prediction, and design of RNAs using dual graphs, similar to our tools developed for tree graphs [35,41,46] (see Discussion below).

### 3.2. Submotifs in Ribosomal RNAs

The ribosome catalyzes protein translation in one of the most conserved biological pathways. Ribosomal RNAs (rRNA) are responsible for many of the functional activities and structural properties of the ribosome. Numerous tertiary interactions in rRNAs, including pseudoknots, dictate the RNA chain folding onto its 3D structure [56]. The 2D and 3D structures of rRNAs are found to be more highly conserved than sequence [57,58]. Moreover, rRNA core is conserved over the phylogenetic tree. To explore whether dual graph representations can help understand 2D structures of rRNAs across different species and identify common submotifs that are conserved, we studied the graph partitioning of rRNAs for small and large ribosomal subunits of various prokaryotic and eukaryotic species (selected from the results obtained in Section 3.1). See Tables S5 and S6 in the Supplementary data for details on the different species and the corresponding PDB files used.

**Figure 7:**
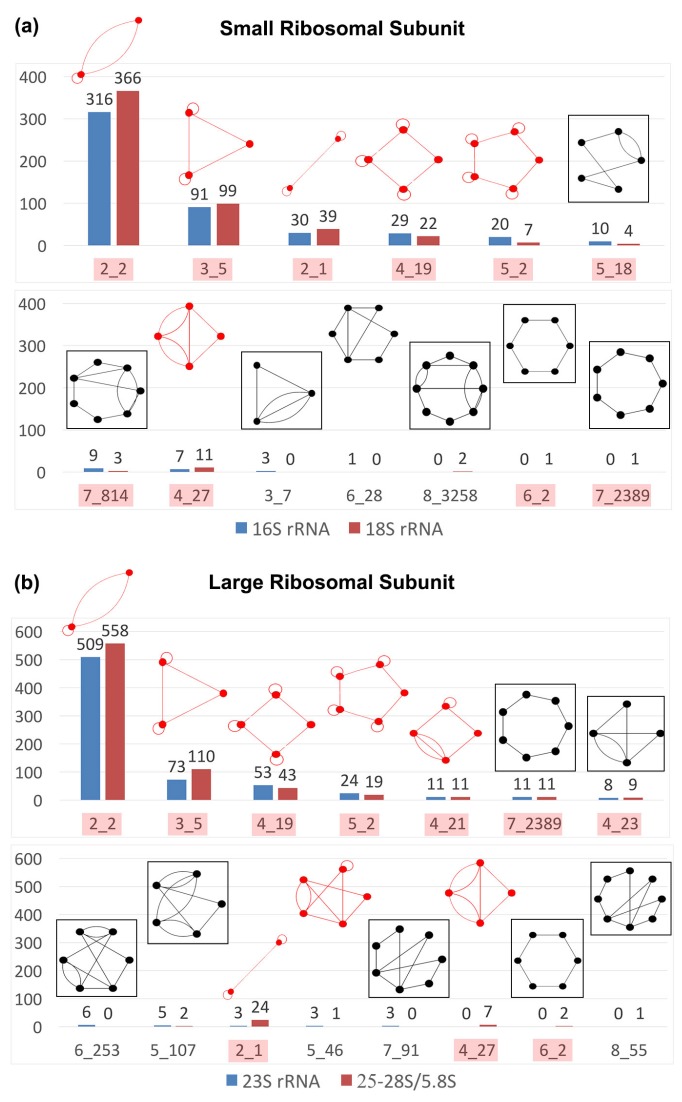
Subgraphs in ribosomes. Dual graph topologies that emerge as subgraph blocks in the (**a**) small (16S and 18S ribosomal RNAs (rRNAs)) and (**b**) large (23S and 25–28S/5.8S rRNAs) ribosomal subunits of various prokaryotic and eukaryotic species (see Tables S5–S6 in the Supplementary data for PDB files used). Subgraph IDs highlighted in red are also common subgraphs shown in Figure 5 and Figure 6. Subgraphs with a black box are unique to rRNA structures in the entire representative RNA structure dataset.

Figure 7 shows the distribution of dual graph blocks in rRNAs of the small and large ribosomal subunits. For the small ribosomal rRNAs, we used 16S rRNA structures from 10 prokaryotic species (three archaea and seven bacteria), and 18S rRNA structures from 12 eukaryotic species (three protozoa, one fungus, three yeast, one plant, one fly, and three mammals). For the large ribosomal subunit, we used 23S rRNA structures from 11 prokaryotic species (three archaea and eight bacteria), and 25-28S/5.8S rRNA structures from 11 eukaryotic species (three protozoa, one fungus, two yeast, one plant, one fly, and three mammals). Apart from the most common subgraph blocks (like 2_2, 3_5, 4_19, 5_2, and 6_2), there is not much in common between the dual graph blocks found in the small and the large ribosomal subunits. Interestingly, the only pseudoknot containing subgraph common between the small and large subunit rRNAs is 4_27 (Figure 7).

Not surprisingly, some of the most common submotifs found in the entire dataset are also the most common ones found in rRNAs (also see Table S7 in Supplementary data for occurrences of non-separable dual graph blocks for the 660 of the 863 2D structures after removing rRNA or rRNA fragment files). However, almost all topologies that emerge only as subgraphs and are present in rRNAs (colored and boxed in black in Figure 7) are not present in non ribosomal RNAs in the representative RNA dataset. For example, all 30 occurrences of the dual graph topology 7_2389 (a 7-way junction) in the representative RNA dataset are found in ribosomal structures; subgraphs 7_814 and 5_18 in the RNA representative dataset are also present in ribosomal RNAs. This suggests that, while ribosomes contain many common submotifs found in other RNA structures, there may be some submotifs that are unique to ribosomal RNAs.

**Figure 8:**
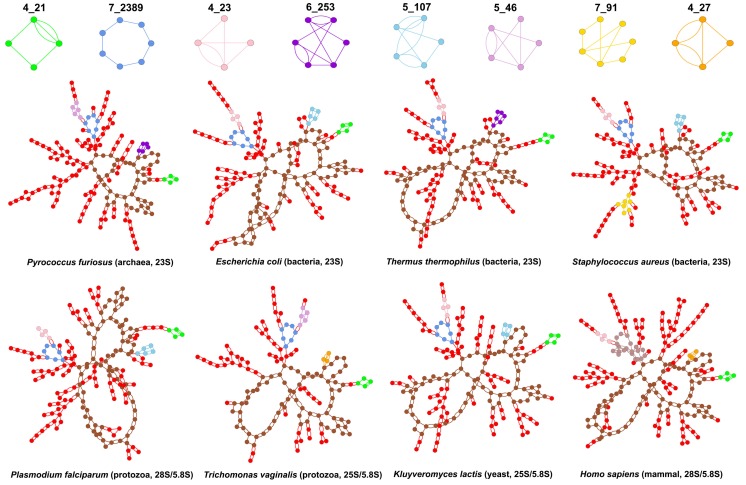
Dual graphs for large subunit rRNAs. Dual graphs corresponding to the 23S (prokaryotes) and 25–28S/5.8S (eukaryotes) rRNAs of large ribosomal subunits for a few representative species. Different colors highlight different subgraph blocks. The subgraph blocks with > 40 vertices are shown in brown. The 21-vertex subgraph in *Homo sapiens* in shown in light brown. Smaller subgraphs that occur in large numbers (2_2, 3_5, 4_19, 5_2, and 2_1) are all colored red and not highlighted separately.

Figure 8 shows the dual graphs for *large* subunit rRNAs of a few representative species, with interesting subgraphs highlighted. The subgraph 4_21 (3-way junction with a pseudoknot shown in green) is common to all prokaryotic and eukaryotic large subunit rRNAs and occurs once in every structure. The subgraph 7_2389 (7-way junction shown in blue) is also present in all large subunit rRNAs, except that of *Homo Sapiens*. Instead, the 28S/5.8S rRNA of *Homo sapiens* contains a larger 21-vertex subgraph (shown in light brown). The subgraph 4_23 (pseudoknot between two internal loops shown in pink) is present in all bacterial 23S rRNAs but not in any archaea, and all eukaryotic 25-28S/5.8S rRNAs except 1 of the protozoa *Trichomonas vaginalis* and the fungus *Thermomyces lanuginosus*. Instead, the archaea 23S rRNAs contain the subgraph 5_46 (shown in light purple), and so does the 25S/5.8S rRNA of the protozoan. The subgraph 6_253 (shown in dark purple) is present in all archaea and three bacteria (*Mycobacterium smegmatis*, *Mycobacterium tuberculosis*, and *Thermus thermophilus*). A smaller pseudoknot subgraph 4_27 (shown in orange) is present in all eukaryotes except the protozoan *Plasmodium falciparum*, the fungus *Thermomyces lanuginosus*, and the yeast *Kluyveromyces lactis*. Interestingly, the subgraph 5_107 (shown in light blue) is present in all species that do not contain either the 6_253 or the 4_27 subgraph, except the fungus.

**Figure 9:**
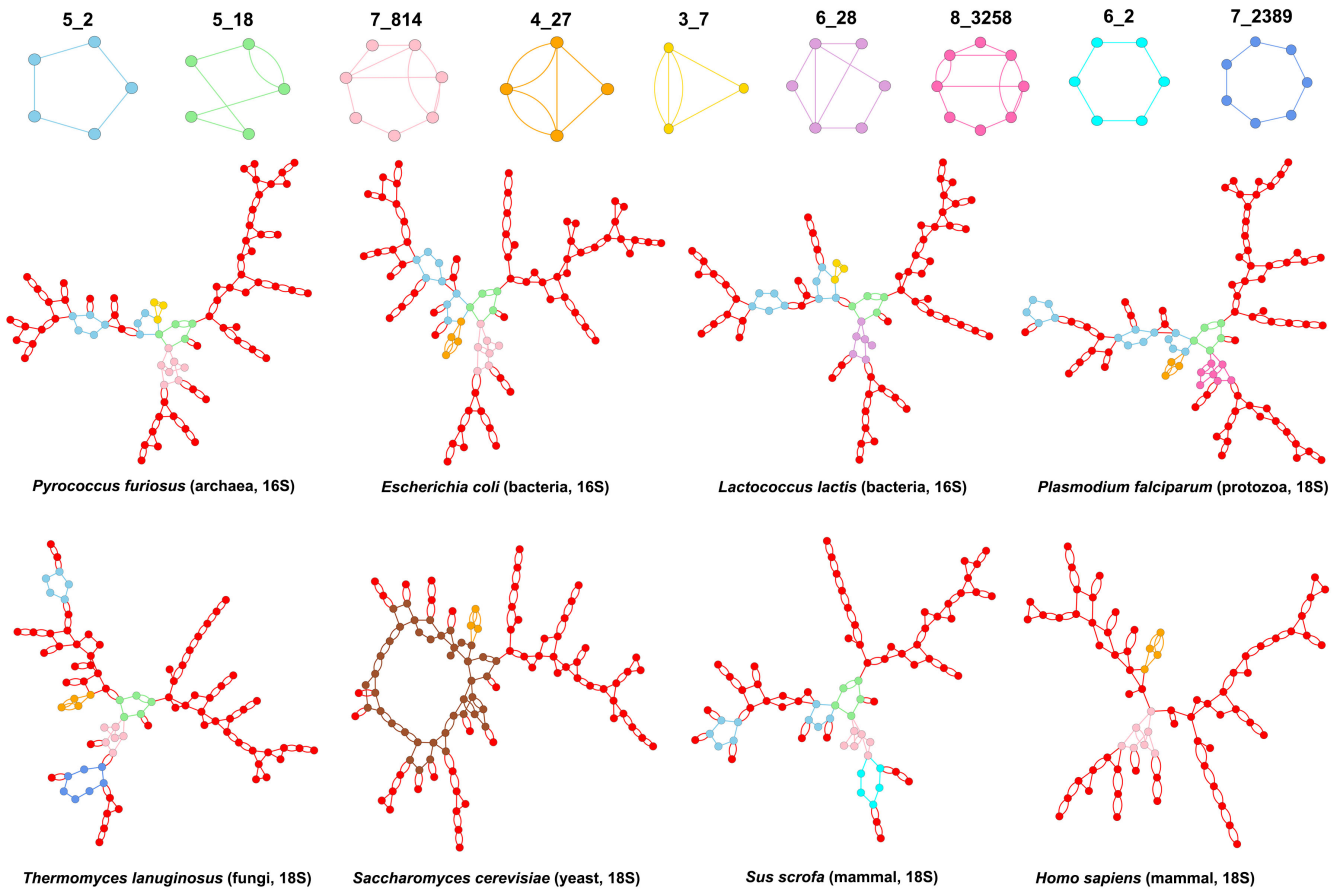
Dual graphs for small subunit rRNAs. Dual graphs corresponding to the 16S (prokaryotes) and 18S (eukaryotes) rRNAs of small ribosomal subunits for a few representative species. Different colors highlight different subgraph blocks. The subgraph block with 41 vertices in the dual graph of *Saccharomyces cerevisiae* is highlighted in brown. Smaller subgraphs that occur in large numbers (2_2, 3_5, 2_1, and 4_19) are all colored red and not highlighted separately.

Figure 9 shows the dual graphs for *small* subunit rRNAs of a few representative species, with interesting subgraphs highlighted. The subgraph 5_2 (5-way junction shown in light blue) is present twice in all prokaryotic 16S rRNAs, and subgraph 5_18 (4-way junction with pseudoknot shown in green) is present once. The seven occurrences of 5_2 and 4 occurrence of 5_18 in eukaryotic 18S rRNAs are distributed over four structures (two of the three protozoa *P. falciparum* and *T. vaginalis*, the fungus *T. lanuginosus*, and one of the three mammals *Sus Scrofa*) and are absent in all the yeast, fly, and plant structures. The subgraph 7_814 (with two junctions and a pseudoknot shown in pink) is present in all but one of the 16S rRNAs (bacterium *Lactococcus lactis* that contains the subgraph 6_28 instead (shown in light purple)), and only in three 18S rRNAs (the fungus *T. lanuginosus* and two mammals *S. scrofa* and *Homo sapiens*); two of the protozoan 18S rRNAs, *P. falciparum* and *T. vaginalis*, contain the subgraph 8_3258 instead (shown in hotpink). A smaller subgraph with a 3-way junction and a pseudoknot, 4_27 (shown in orange), is present in 11 of the 12 18S rRNAs (except the mammal *S. scrofa*), and 7 of the 10 16S rRNAs. Interestingly, the 16S rRNAs that do not contain 4_27 (2 archaea *Methanocaldococcus jannaschii* and *Pyrococcus furiosus*, and bacterium *L. lactis*) contain a smaller pseudoknot subgraph 3_7 (shown in yellow). All 16S rRNAs and five of the 18S rRNAs consist of only subgraphs with 2–8 vertices (i.e., no subgraphs larger than eight vertices exist in their 2D structures). Interestingly, these five 18S rRNAs are the ones mentioned above that stand out from other 18S rRNAs.

## 4. Discussion

Representing RNAs using a coarse-grained approach and characterizing RNA modular units can help shed insights into structure/function relationships among RNAs. Partitioning RNAs can similarly help analyze recurring 2D submotifs and suggest functional relationships. Application of our partitioning method to large RNA structures such as rRNAs could help detect evolutionary pathways. Because the partitioning is coarse-grained, it provides a clear visualization of submotifs even for very large and complex structures like ribosomes. Our graph representations also provide additional flexibility in terms of RNA size as they focus on the overall topology and connectivity of RNA 2D structural elements, rather than the exact number of residues in stems and loops that can change even in related structures. This flexibility also allows us to ignore small variations in base pairing in RNA 3D structures as detected by different 2D structure annotation methods. As long as the number of stems and their connectivity remains the same, the dual graph topology will be the same. However, differences or errors in base pair annotations that lead to fewer or more stems and loops (especially true for stems and pseudoknots that involve only a few residues) may lead to a different dual graph.

One main advantage of using dual graphs for RNA 2D structures is their capability of representing pseudoknots. Combined with our dual graph partitioning algorithm that keeps pseudoknots intact, our method offers an effective tool to study RNA structures as well as submotifs and substructures containing pseudoknots. Although the partitioning of structures in the representative RNA dataset reveals many submotifs with pseudoknots, only a few of them occur frequently. In addition, almost all subgraph blocks contain 2–9 vertices (5332 of the 5380 subgraph blocks). Increasing our dual graph library for more than nine vertices and applying dual graph partitioning on more RNAs may certainly increase this submotif library. However, our results suggest that RNAs adopt a combination of only a limited number of submotifs of all theoretically possible motifs, which may simplify the study of RNA substructures and design.

Various extensions can be envisioned for our work with dual graph representations. Similar to our work with tree graphs [38], extending the dual graph representation to represent 3D RNA structures as 3D dual graphs will allow us to sample dual graph conformations. Enumerating dual graphs with more than nine vertices and annotating all dual graph topologies as either “existing”, “RNA-like” or “non RNA-like” (as done previously for both tree and dual graphs by clustering [34,36,42]) will also be crucial. We are also working to modify our current partitioning methodology to differentiate between non-recursive and recursive pseudoknot blocks; recursive pseudoknot blocks can be further partitioned (with the new partitioning methodology being developed) into pseudoknot-free structure and/or isolated pseudoknots [59]. Also useful for RNA submotif search is annotating the pseudoknot containing subgraphs and corresponding substructures based on types of loop residues involved (i.e., hairpins, internal loops, or junctions [60,61]) (see below).

**Figure 10:**
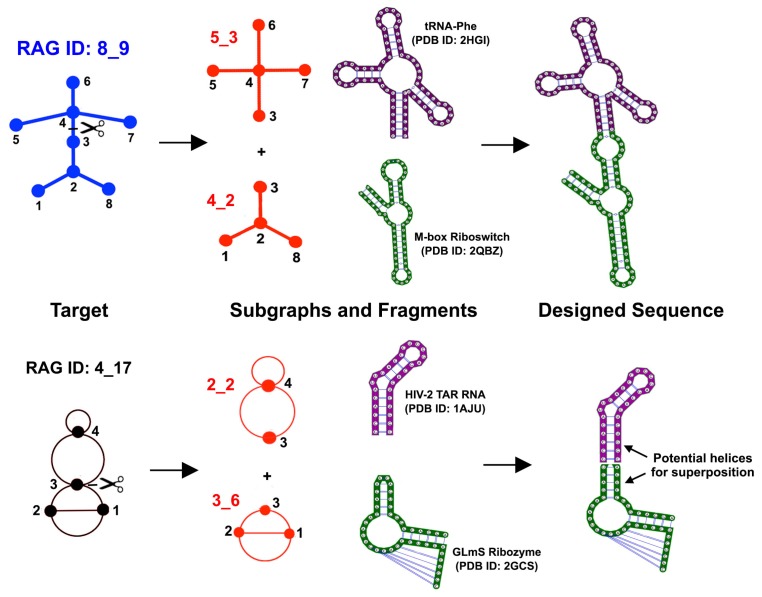
Proposed design pipeline for dual graphs. Fragment assembly for design of novel RNA topologies for tree and dual graphs using the RNA-As-Graph (RAG) subgraph and atomic fragment libraries. The tree graph design results are taken from [35], and a similar pipeline is proposed for dual graphs.

The RAG-3Dual database (available online in [49]) we have assembled in this study can be extended to include all possible subgraphs up to nine vertices (not just basic non-separable blocks) as well as submotifs from all RNA containing 3D structures (available from PDB) to provide a comprehensive database of RNA substructures. Such a library of structures, combined with the 3D dual graph representation, will allow us to develop a search tool for RNA structures and substructures similar to an RAG-3D search tool developed for tree graphs [46]. Furthermore, our fragment assembly algorithm F-RAG [35,41] can be extended to work with dual graph representations. As shown in Figure 10, to design sequences to fold onto novel tree graph topologies (e.g., the 8_9 topology as shown), the target graph is first partitioned into subgraphs (e.g., 5_3 and 4_2 as shown), and the fragments corresponding to those subgraphs (from the RAG-3D database for tree graphs) are combined using residues in the common loop and flanking stems. Similarly, the target dual graph (e.g., the 4_17 topology as shown) can be partitioned into subgraphs (e.g., 2_2 and 3_6 as shown), and the fragments corresponding to those subgraphs from the RAG-3Dual database can be assembled together using common stems. This will facilitate the prediction and design of more complex RNA structures with pseudoknots.

## 5. Conclusions

We have presented an efficient and systematic way of identifying modular units of RNA 2D structures based on dual graph partitioning. Using our dual graph representation and partitioning algorithm on a representative set of RNA structures, we identify 94 dual graph topologies (between two and nine vertices) that correspond to existing RNA structures, and 56 dual graph topologies (between two and nine vertices) that emerge as non-separable subgraph blocks (Figure 4). Of the 56, 29 topologies (shown in black in Figure 4) correspond to subgraphs only and are not part of 94 existing dual graph topologies. The most frequent motifs are shown in Figure 5 (pseudoknot-free) and Figure 6 (with pseudoknots). We have extracted 3D atomic fragments for all examples corresponding to these subgraph blocks from our representative dataset, and cataloged them based on their dual graph IDs in a dataset of RNA 3D substructures called RAG-3Dual. The existing 94 topologies and the RAG-3Dual dataset are available online [49]. Our partitioning method operates in a biologically meaningful way that keeps junctions and pseudoknots intact. This allows us to study motifs in structures with pseudoknots, including complex RNA structures like ribosomes, as reported here. Further developments for the prediction and design of RNAs with pseudoknots can be envisioned.

References

## Supplementary Materials

The following are available online: http://www.mdpi.com/2073-4425/9/8/371/s1. Table S1: 94 dual graph topologies between 2–9 vertices, with IDs and number of structures, for the representative RNA structure dataset. Table S2: Number of structures in the representative dataset that have dual graphs with ≥10 vertices. Table S3: Dual graph topologies that were removed from the updated library of existing dual graph topologies. Table S4: Number of subgraph blocks ≥10 vertices identified by the dual graph partitioning algorithm in the representative dataset of RNA structures. Table S5: PDB IDs and chains for rRNAs of small ribosomal subunits for 10 prokaryotic and 12 eukaryotic species. Table S6: PDB IDs and chains for rRNAs of large ribosomal subunits for 11 prokaryotic and 11 eukaryotic species. Table S7: Occurrences of non-separable dual graph blocks between 2–9 vertices in 660 of the 863 RNA 2D structure files without rRNA residues. Supplementary Material S2: Description of the RNA-As-Graphs dual graph resource.
